# Complete mitochondrial genome of darkfin hind *Cephalopholis urodeta* (Perciformes, Epinephelidae)

**DOI:** 10.1080/23802359.2016.1214542

**Published:** 2016-12-09

**Authors:** Minglan Guo, Hui Huang, Yongli Gao

**Affiliations:** a Key Laboratory of Tropical Marine Bio-Resources and Ecology, South China Sea Institute of Oceanology, Chinese Academy of Sciences, Guangzhou, China;; b Tropical Marine Biological Research Station in Hainan, Chinese Academy of Sciences, Sanya, China;; c The Center of Instruments and Determination, South China Sea Institute of Oceanology, Chinese Academy of Sciences, Guangzhou, China

**Keywords:** Grouper, mitogenome, genetics

## Abstract

Darkfin hind, *Cephalopholis urodeta,* belongs to the subfamily Epinephelinae. It is one of the most important fish species in coral-reef ecosystem. In this study, the complete mitochondrial (mt) genome of *C. urodeta* has been determined. It was 16,592 bp in length and contained 13 protein-coding genes, 2 ribosomal RNA genes, 22 transfer RNA genes and 2 non-coding regions. The mitogenome sequence of *C. urodeta* shared 94% and 92% similarity to that of *C. sonnerati* and *C.sexmaculata*, respectively. Phylogenetic tree was made based on the concatenated sequences of 12 protein-coding genes on mtH-strand. All the results provide insights into the evolution in the subfamily Epinephelinae.

Groupers are bottom-associated fishes found in the tropical and subtropical waters of all oceans. Darkfin hind, *Cephalopholis urodeta,* is one of the common coral reef species of grouper found in outer reef areas, lagoons and back-reef areas and on the reef-top. It is a widespread species occurring at the tropical islands and shallow banks of the Indian and west-central Pacific Oceans, and the northern coast of Australia. Because of its small size (9–21 cm standard length), *C. urodeta* is not of much interest as a food fish (Heemstra & Randall 1993). However, *C. urodeta* shows subsistence commercial status (Tyler et al. 2009) and important ecological functions because it is one of the major predators feeding on a variety of fishes, crustaceans and cephalopods in coral-reef ecosystem (Randall & Brock 1960; Heemstra & Randall 1993; Pinault et al. 2014). Some confusions and disagreements remain puzzled on the classification and nomenclature of this species by morphological analysis (Allen & Steene 1988; Heemstra & Randall 1993).

In this study, three individuals of *C. urodeta* were obtained from Triton island (15°47′N 111°12′E) of China and species identifications were performed according to FAO Groupers of the World (Heemstra & Randall 1993). Dorsal muscle (Disposition number: ZJ201507A-C) were collected from frozen fishes. This study involving animals was carried out in accordance with the recommendations of “Animal Care and Ethical Committee, South China Sea Institute of Oceanology, Chinese Academy of Sciences.” Total genomic DNA was isolated from tissue samples of dorsal muscle using standard phenol-chloroform extraction and ethanol precipitation methods. The complete mitochondrial (mt) genome of *C. urodeta* was obtained with long PCR approach. Primers used were designed on the basis of aligned mitogenome sequences of *C. sonnerati* (KC593378.1), *C. argus* (KC593377.1), *C. boenak* (KC537759.1) and *C. sexmaculata* (KJ469385.1).

The complete mtDNA sequence of *C. urodeta* (GenBank accession number: KU891818) was 16,592 bp in length, consisting of 13 protein-coding genes, 22 tRNA genes, two rRNA genes, and two non-coding regions: origin of light-strand replication (O_L_) and control region (CR or D-loop) (Table 1). Most of the genes were encoded on the heavy strand (H strand) except for ND6 and eight tRNA genes (*tRNA^Gln^, tRNA^Ala^, tRNA^Asn^, tRNA^Cys^, tRNA^Tyr^, tRNA^Ser(UCN)^, tRNA^Glu^* and *tRNA^Pro^*), which are encoded on the L-strand. All genes showed the typical gene arrangement conforming to the vertebrate consensus (Johansen et al. 1990; Boore 1999). Sequence overlaps were showed between protein-coding genes, including ATP8-ATP6, ATP6-COIII, NDL4-ND4, and ND5-ND6, and/or tRNA genes, such as *tRNA^Ile^*- *tRNA^Gln^*, *ND2*- *tRNA^Trp^*, *COIII*- *tRNA^Gly^*, and *tRNA^Tyr^*- *tRNA^Pro^*. The 40 bp fragment of O_L_, as in most vertebrates, overlapped the *tRNA^Cys^* gene by 1 bp and was located in a cluster of five tRNA genes (WANCY region; Table 1) between the *tRNA^Asn^* and *tRNA^Cys^*. The other non-coding region CR was bound by *tRNA^Pro^* and *tRNA^Phe^*. Overall base composition of the mitogenome was estimated to be 29.47% A, 28.36% C, 15.99% G and 26.18% T, respectively, with a high A + T content (55.64%), indicating an obvious anti-guanine bias commonly observed in fishes (Cantatore et al. 1994; Wang et al. 2008). The mitogenome sequence of *C. urodeta* showed 94% and 92% identity to that of *C. sonnerati* and *C. sexmaculata*, respectively.

**Table 1.** Characteristics of the mtgenome of *C. urodeta*.

	Size		Codon				
Locus	Nucleotide (Position)	Amino acid	Start	Stop	Anti-codon	Intergenic nucleotide^a^	Strand^b^
*tRNA^Phe^*	70 (1–70)				GAA	0	H
*12S rRNA*	957 (71–1027)					0	H
*tRNA^Val^*	71 (1028–1098)				TAC	1	H
*16S rRNA*	1715 (1100–2814)					1	H
*tRNA^Leu(UUR)^*	75 (2815–2889)				TAA	0	H
*ND1*	975 (2890–3864)	324	ATG	TAA		6	H
*tRNA^Ile^*	70 (3871–3940)				GAT	−2	H
*tRNA^Gln^*	71 (4009–3939)				TTG	0	L
*tRNA^Met^*	69 (4010–4078)				CAT	0	H
*ND2*	1047 (4079–5124)	348	ATG	TA-		0	H
*tRNA^Trp^*	71 (5125–5195)				TCA	1	H
*tRNA^Ala^*	69 (5265–5197)				TGC	0	L
*tRNA^Asn^*	73 (5338–5266)				GTT	0	L
O_L_	40 (5339–5378)					−1	–
*tRNA^Cys^*	68 (5445–5378)				GCA	0	L
*tRNA^Tyr^*	71 (5516–5446)				GTA	1	L
*COI*	1551 (5518–7068)	516	GTG	TAG		0	H
*tRNA^Ser(UCN)^*	71 (7139–7069)				TGA	3	L
*tRNA^Asp^*	73 (7143–7215)				GTC	8	H
*COII*	691 (7224–7914)	230	ATG	T–		0	H
*tRNA^Lys^*	73 (7915–7987)				TTT	1	H
*ATP8*	168 (7989–8156)	55	ATG	TAA		−10	H
*ATP6*	684 (8147–8830)	227	CTG	TAA		−1	H
*COIII*	786 (8830–9614)	261	ATG	TA-		0	H
*tRNA^Gly^*	72 (9615–9686)				TCC	0	H
*ND3*	349 (9687–10,035)	116	ATG	T–		0	H
*tRNA^Arg^*	69 (10,036–10,104)				TCG	0	H
*ND4L*	297 (10,105–10,401)	98	ATG	TAA		−7	H
*ND4*	1381 (10,395–11,775)	460	ATG	T–		0	H
*tRNA^His^*	70 (11,776–11,845)				GTG	0	H
*tRNA^Ser(AGY)^*	75 (11,846–11,920)				GCT	9	H
*tRNA^Leu(CUN)^*	73 (11,930–12,002)				TAG	0	H
*ND5*	1839 (12,003–13,841)		ATG	TAA		−4	H
*ND6*	522 (14,359–13,838)	173	ATG	TAA		0	L
*tRNA^Glu^*	69 (14,428–14,360)				TTC	4	L
*Cyt b*	1141 (14,433–15,573)	377	ATG	T–		0	H
*tRNA^Thr^*	73 (15,574–15,646)				TGT	−1	H
*tRNA^Pro^*	70 (15,715–15,646)				TGG	0	L
*D-loop*	877 (15,716–16,592)						–

^a^Numbers correspond to the nucleotides separating different genes. Negative numbers indicate overlapping nucleotides between adjacent genes.

^b^H and L indicate genes transcribed on the heavy and light strands, respectively.

Most of the grouper mtprotein-coding genes began with the typical start codon ATG. As in many other metazoans (Wolstenholme 1992), the COI gene began with GTG in *C. urodeta*. Different from most other teleosts and basal groupers (Craig & Hastings 2007; Zhuang et al. 2013), CTG was the start codon of the ATP6 gene in *C. urodeta* (Table 1). The protein-coding genes COII, ND3, ND4 and Cyt b were all terminated with the incomplete stop codon T–, while ND2 and COIII were TA- (Table 1). It was completed with the addition of 3′ adenine residues to the mRNA by post-transcriptional polyadenylation (Ojala et al. 1981; Coucheron et al. 2011). The pattern of codon usage in the *C. urodeta* mtDNA is shown in Table 2. There were 3807 codons for all the protein-coding genes after excluding the incomplete stop codons. The concatenated sequences of 12 protein-coding genes on mtH-stand were aligned with codon constraint using Clustal X (http://www.ebi.ac.uk/clustalW/). Phylogenetic tree (Figure 1) was constructed according to the alignment of amino acid sequences with MEGA 4.0 (http://megasoftware.net).

**Figure 1.**
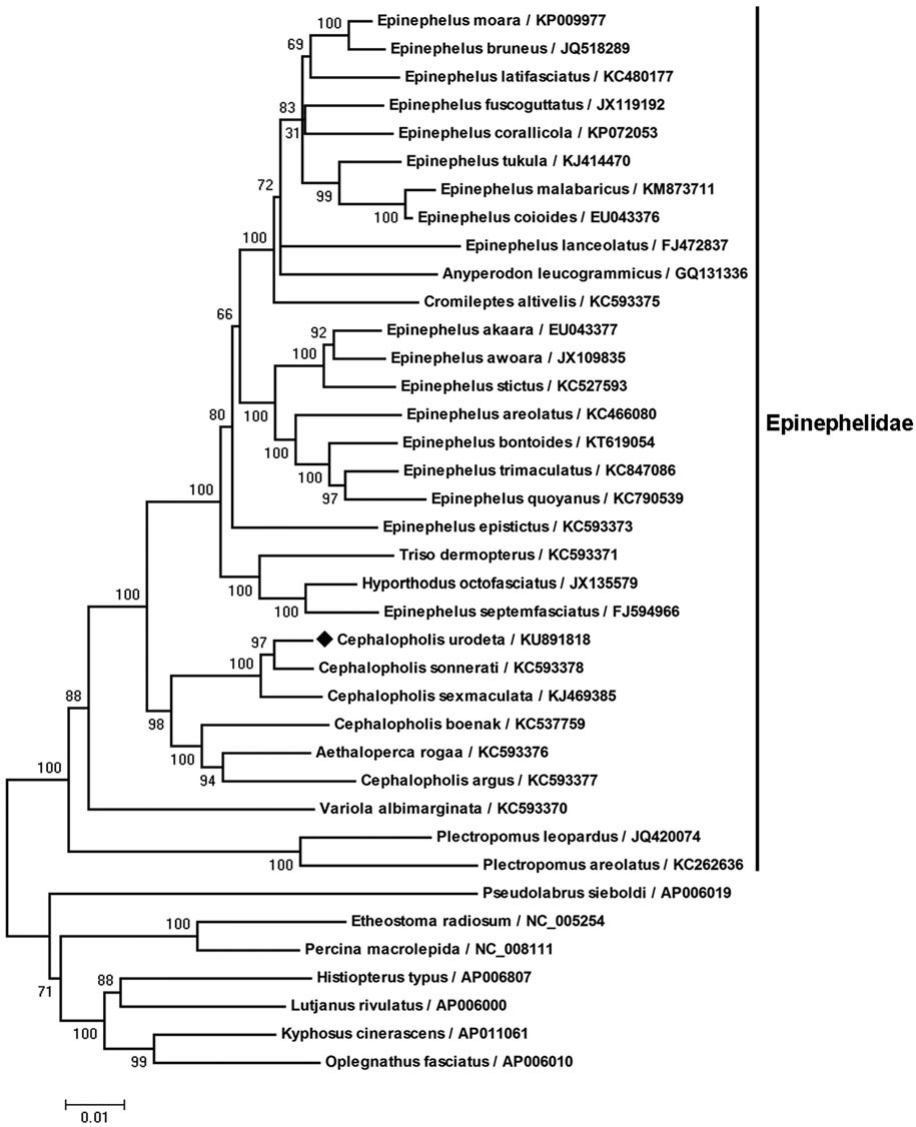
Phylogenetic , tree of *C. urodeta* and other fishes in suborder Percoidei. Phylogenetic tree was constructed according to the alignment of amino acid sequences of 12 protein-coding genes on mtH-strand by the neighbour-jointing method within MEGA 4.0 performing 1000 replications of bootstrap. The bootstrap values were indicated at the nodes of the tree. NCBI RefSeq or GenBank accession number of each species was listed on the right of the species name. *Cephalopholis urodeta* was clustered into the branch of family Epinephelidae.

**Table 2.** Codon usage of the protein-coding genes in *C. urodeta* mtgenome.

Amino acid	Codon	No.	%	Amino acid	Codon	No.	%
Phe	UUU	80	2.10	Stop	UAA	6	0.21
	UUC	158	4.15		UAG	1	0.03
Leu	UUA	94	2.47	His	CAU	30	0.79
	UUG	12	0.32		CAC	76	2.00
	CUU	137	3.60	Gln	CAA	93	2.44
	CUC	126	3.31		CAG	7	0.18
	CUA	241	6.33	Asn	AAU	42	1.10
	CUG	33	0.87		AAC	78	2.05
Ile	AUU	144	3.78	Lys	AAA	73	1.92
	AUC	130	3.41		AAG	4	0.11
Met	AUA	100	2.63	Asp	GAU	31	0.81
	AUG	63	1.65		GAC	49	1.29
Val	GUU	58	1.52	Glu	GAA	82	2.15
	GUC	56	1.47		GAG	13	0.34
	GUA	96	2.52	Cys	UGU	13	0.34
	GUG	13	0.34		UGC	14	0.37
Ser	UCU	39	1.02	Trp	UGA	104	2.74
	UCC	74	1.94		UGG	13	0.34
	UCA	68	1.79	Arg	CGU	12	0.32
	UCG	7	0.18		CGC	10	0.26
Pro	CCU	48	1.26		CGA	47	1.23
	CCC	88	2.31		CGG	9	0.24
	CCA	75	1.97	Ser	AGU	4	0.11
	CCG	7	0.18		AGC	46	1.21
Thr	ACU	46	1.21		AGA	***	—
	ACC	115	3.02		AGG	***	—
	ACA	129	3.39	Gly	GGU	53	1.39
	ACG	13	0.34		GGC	74	1.94
Ala	GCU	84	2.21		GGA	88	2.31
	GCC	128	3.36		GGG	27	0.71
	GCA	130	3.41		NNA^a^	1426	37.49
	GCG	7	0.18		NNT^a^	860	22.58
Tyr	UAU	39	1.02		NNC^a^	1292	33.92
	UAC	70	1.84		NNG^a^	229	6.01

A total of 3807 codons were analyzed excluding the incomplete stop codons.

^a^ Amount and percentages of codons with the 3rd site nucleotide composition of A, T, C, G.

*** the stop code AGA and/or AGG (instead of Ser) was not detected.

The 12S rRNA and 16S rRNA genes lied between tRNA^phe^ and tRNA^Val^, and tRNA^Val^ and tRNA^Leu (UUR)^, respectively. A moderate nucleotide compositional bias, A (32.71%) >C (25.11%) >T (21.22%)>G (20.96%), was found in rRNA genes of *C. urodeta*. The tRNA genes ranged in size from 68 to 75 bp. Two forms of tRNA^Leu^ (UUR and CUN) and tRNA^Ser^ (UCN and AGY) were contained in the mtgenome of *C. urodeta* (Table 1). Most tRNAs could be folded into the typical clover-leaf secondary structure by tRNAscan-SE (http://lowelab.ucsc.edu/tRNAscan-SE/). However, tRNA^Ser (AGY)^ was found to lack the entire dihydrouridine (DHU) arm, reducing its secondary structure to a ‘truncated cloverleaf’. Similar phenomena have been reported in groupers (Zhuang et al. 2013) and most metazoans (Garey & Wolstenholme 1989). Aligning with sequences from other grouper species, CR of *C. urodeta* contained with three domains: the extended termination associated sequences (ETAS), central conserved domain (CCD), and conserved sequence blocks (CSB). The motif-TACAT and reversed motif-ATGTA were observed in the ETAS domains. Both motifs could form stable hairpin loops which presumably act as sequence-specific signals for termination of mtDNA replication (Saccone et al. 1991). All the data would contribute to the genetic conservation, species identification and phylogeny analysis of Epinephelinae.
